# Childhood Infections and Trauma as Risk Factors for Stroke

**DOI:** 10.1007/s11886-014-0527-y

**Published:** 2014-08-17

**Authors:** Elena Moraitis, Vijeya Ganesan

**Affiliations:** 1 Rheumatology/Infectious Diseases and Immunity Unit UCL Institute of Child Health and Rheumatology Department, Great Ormond Street Hospital for Children, London, UK; 2 Neurosciences Unit UCL Institute of Child Health, London, UK, Neurology Department, Great Ormond Street Hospital for Children, London, UK

**Keywords:** Arterial ischemic stroke, Cerebral arteriopathy, Pediatric, Risk factor, Childhood, Infection, Trauma

## Abstract

Stroke is as common as brain tumor in children. The etiology of childhood arterial ischemic stroke (AIS) appears to be multifactorial, resulting from the interaction between genetic predisposition and environmental triggers. The risk factors for AIS in children are markedly different from the atherosclerotic risk factors in adults. Trauma and infections have been identified as associations in previous studies and are exposures of particular interest because of their increased prevalence in the children. The aim of this review article is to provide an overview of the research studies that have addressed the role of infections and trauma in pediatric AIS.

## Introduction

Pediatric stroke (acute brain injury from a vascular cause) affects as many children as brain tumor; two-thirds of survivors experience impairments across a range of domains, with lifelong health, personal, and societal consequences [1]. The subtypes of childhood stoke are ischemic stroke (arterial ischemic stroke) and cerebral sinovenous thrombosis), and nontraumatic intracranial hemorrhage; incidence of these is roughly equal [2]. Advances in acute treatment and prevention (both primary and secondary) of childhood stroke have been hampered by limited understanding of the underlying disease mechanisms. Specifically, atheromatous cerebrovascular disease, the commonest disease mechanism in adults, is not implicated in children, thus, distinct treatment approaches are likely to be needed. A current view is that childhood AIS is likely to have a multifactorial etiology that involves interplay between host vulnerability (possibly because of genotype) and environmental insults [3, 4•]. In this review, we will focus on 2 common environmental factors, infection and trauma, and discuss their potential role in the genesis of childhood AIS.

## Risk Factors for Arterial Ischemic Stroke in the Children Population—an Evolving Understanding

Arterial ischemic stroke (AIS) can be defined as an acute focal neurologic syndrome because of cerebral infarction in an arterial distribution. The risk factors for arterial ischemic stroke (AIS) in children markedly differ from those in adults [5–7]. A large proportion of children with AIS have another medical diagnosis that predisposes them to stroke, for example sickle cell disease, congenital heart disease, acute systemic diseases or a range of genetic disorders [5, 6, 8, 9•]. In addition, comprehensive investigation has identified a large number of factors that are associated with childhood AIS. The term ‘risk factor’ has been widely used in the literature to describe these factors. However, in the absence of case-control data, direct causality remains unproven in many instances and it has been suggested that these risk factors are ‘presumptive’ rather than ‘definite’ [8].

Studies of large cohorts have provided complementary data on presumptive risk factors for childhood AIS. For example, a study of >200 children from our institution identified arteriopathies in >80 % and highlighted a potential role for trauma and previous chickenpox in children who had been healthy prior to AIS [6]. Data from a German registry [10] identified a rather lower rate of arteriopathy (18 %, although the extent of evaluation was unclear) and a markedly higher rate of thrombophilia—likely reflecting the genetic makeup of that population. AIS was attributed to infection in around 10 % of cases. Of note, arteriopathy was significantly associated with AIS recurrence.

The International Pediatric Stroke Study (IPSS) is an international collaboration that has enabled analyses of the causes and consequences of stroke in large cohorts of children. Although hampered by variability in the clinical approach and completeness of investigation in individual collaborating centers, the strength of the IPSS is the large multicenter international dataset that is available for interrogation. In an analysis of AIS risk factors in 676 children from 30 centers in 10 countries, the major categories that emerged were arteriopathies (in >50 %), infection (in around 25 %) cardiac disorders, acute head and neck disorders, acute systemic conditions (such as sepsis and dehydration), and chronic systemic conditions (eg, sickle cell disease and connective tissue disorders). In contrast, risk factors for atheroma were rare (in <2 %). Of note, risk factors were identified in >90 % of cases.

These observations were recently confirmed in a population-based study of childhood stroke from the south of England (an area that contains around 50 % of the UK pediatric population). Acute systemic illnesses and arteriopathy were the two leading risk factor categories identified. Infection defined as recent infection, acute systemic disorders or acute or chronic head and neck disorders, was reported at a total frequency of 28 %. Taken together, a common theme that emerges from these studies is the high rate of arteriopathy and infection observed in children with AIS.

## Cerebral Arteriopathies in Childhood AIS

As previously stated, arteriopathies implicated in childhood AIS are nonatheromatous. They are currently defined and categorized on the basis of radiological appearances, usually on the basis of magnetic resonance angiogram (MRA) findings. The majority of abnormalities are intracranial, although up to 25 % may have cervical disease [11]. Specific classification systems have been proposed to promote consistency in terminology, initially by Sebire et al (2004) and more recently the CASCADE system [12]. Specific subtypes of arteriopathy associated with childhood AIS include moyamoya (occlusive disease of the terminal internal carotid arteries with basal collaterals) and arterial dissection.

The most common arteriopathy described in the context of pediatric AIS is focal occlusive disease of the terminal internal carotid or proximal middle/anterior cerebral artery. This was initially termed transient cerebral arteriopathy (TCA) [13]. This is radiologically characterized by unilateral, focal stenosis typically of terminal internal carotid artery or proximal segments of anterior and/or middle cerebral arteries, showing nonprogression or regression on vascular imaging 6 months after index AIS [14]. The interval of 6 months is arbitrary, and there are cases which show improvement after considerably longer periods [15, 16]. Since many children with AIS are not re-imaged, in 2009 the IPSS proposed that the term focal cerebral arteriopathy of childhood (FCA) [17], defined as previously at presentation, but without the requirement to show temporal evolution with re-imaging.

Many children with chickenpox preceding AIS, and who are thought to have post-*varicella* cerebral infarction, also have this pattern of arteriopathy. It has been proposed that the term post-*varicella* arteriopathy (PVA) is used for these cases and there is confusion in the literature about the degree of overlap between FCA and PVA. In fact, these are radiologically indistinguishable entities, the key difference being the history of antecedent VZV in the PVA cases. An analysis of predictors of arteriopathy in the IPSS dataset identified recent infection, particularly upper respiratory infection, as a strong predictor of FCA [17]. Thus, it is likely that FCA represents a focal inflammatory response to a range of infectious agents (including *varicella* *)* and that the association between infection and arteriopathy in the genesis of childhood AIS is mediated via the effect of infection on the cerebral circulation.

There is controversy about the possible overlap between FCA and childhood primary angiitis of the central nervous system (cPACNS) [18, 19]. The literature contains examples of patients with indistinguishable clinical presentation and radiological features, labeled as either FCA or cPACNS. The difficulty in distinguishing between these two entities arises from the fact that the patients with FCA fulfil the Calabrese et al criteria for cPACNS [20], from the lack of sensitivity of the investigations available for cPACNS and problems associated obtaining tissue for histologic diagnosis. Radiological studies, even catheter angiography, are unlikely to have sufficient resolution to differentiate between an active vs a burnt-out inflammatory process; however, circulating biomarkers of endothelial injury may be more informative in this regard [21]. In current clinical practice there remain major inconsistencies in diagnosis and categorization between FCA and cPACNS, with knock on effects in terms of management.

## Mechanisms Linking Infection and Arteriopathy

Several potential biological mechanisms may explain how infections and inflammation increase the risk of ischemic stroke [22–25]. Infection could contribute to stroke by promoting systemic procoagulant effects and local inflammation (or even direct pathogen invasion) of cervical or cerebral blood vessels [26]. Disturbances of immune-hematological mechanisms occur in the context of infection-associated stroke, including reduced concentrations of circulating antithrombotic protein C, elevated plasma concentrations of C4b-binding protein S (a main inhibitor of the anticoagulant protein S), lower ratio of active tissue plasminogen activator to plasminogen activator inhibitor, and significantly increased fibrin D-dimer concentration, cardiolipin immunoreactivity, and fibrinogen concentrations [23, 24, 27]. Increased peripheral concentrations of C-reactive protein and pro-inflammatory cytokines are associated with systemic infection and can contribute to a procoagulant state by stimulating monocytes to produce tissue factor [28]. Raised concentrations of IL-6 in adult patients with AIS have been linked to decreased levels of free protein S, suggesting that IL-6 can modulate this procoagulant pathway [29]. Infections increase platelet reactivity and platelet-leukocyte interactions, leading to an increased risk of platelet aggregation, potentially precipitating stroke. Platelet activation (assessed by P-selectin expression) and platelet-leukocyte aggregates are both increased in stroke patients [30]. In one study, platelet activation was increased in 21 stroke patients with history of infection within 1 week of stroke, compared with 37 stroke patients with no history of infection [30], and increased platelet activation has also been shown in volunteers with URTI in other study [31]. A study in children has demonstrated that a minor infectious stimulus in childhood relevant to normal daily life is associated with endothelial dysfunction, this raising the possibility that infection may contribute to mechanisms relevant to the development of early atherosclerosis [32].

As a general response to tissue injury, inflammation is ubiquitous [22, 33, 34]. As with other tissues, microbial infection is one trigger for vascular tissue inflammation [35], and inflammatory cells then subsequently destroy bacteria or virally infected host cells. Inflammation is also triggered by tissue trauma not initiated by infection, and this may be relevant when considering the link between trauma and childhood AIS, discussed further below. Ideally, the inflammatory response is short-lived and localized to the site of tissue invasion or trauma. In contrast, inflammation becomes pathogenic when it occurs at an inappropriate site, or is excessive in extent or duration [35]. In this context inflammation is a major contributing factor to many vascular events, including infection-associated stroke and acceleration of progression of vascular disease.

## Infectious Pathogens in Childhood Arterial Ischemic Stroke

The association between acute infections and childhood AIS is supported by recent studies, and a number of infectious pathogens have been linked to stroke in case reports or larger epidemiologic studies.

A recent case-control study nested within a retrospective population based cohort of 2.5 million children enrolled in an integrated health care plan and identified as part of Kaiser Pediatric Stroke Study (KPSS) in Northern California, showed that infections are common independent risk factor for AIS [36]. The study included 126 AIS cases and 378 matched controls, and demonstrated that a minor acute infection in the 4 weeks prior to the stroke episode increased a child’s risk of AIS by 4-fold. Major infections (bacteremia/sepsis within 1 week, and meningitis/encephalitis within 4 weeks prior to infarct) were significantly more prevalent in the AIS group compared with the controls. However, the most common infectious diagnoses among the cases were upper respiratory tract infections, other viral syndromes, acute otitis media, and acute gastroenteritis; no single type of infection predominated. Thus, it appears that relatively minor and common childhood infections are potentially implicated in the pathogenesis of childhood AIS.

## Varicella Zoster (VZV)

VZV is a ubiquitous, exclusively human, DNA virus. After primary infection (chickenpox), the virus becomes latent in the ganglionic neurons and can reactivate manifesting with herpes zoster (shingles). The initial association between childhood AIS and a common childhood infection was in the context of *varicella zoster* *.* In 1999, Sebire et al undertook a case-control study comparing 11 children with AIS to 44 healthy controls. 64 % of children in the AIS group had *varicella* at a median of 6 weeks prior to the stroke, vs 9 % in the control group [37]. In 2001, a Canadian group reported that 1 in 3 cases of AIS followed *varicella* in a prospective cohort study of 70 consecutive children with AIS recruited at 2 institutions [38]. Recently, 2 large studies have brought further confirmatory evidence on the connection between VZV and childhood AIS. Thomas et al undertook a self-controlled case series analysis of data from 4 general practice databases and found a 4-fold increased risk of stroke in children within 6 months of chickenpox [39]. A recent retrospective cohort study of a large number of herpes zoster cases and matched controls found that herpes zoster is an independent risk factor for vascular disease under 40 years of age in the UK population [40].

**Fig. 1:**
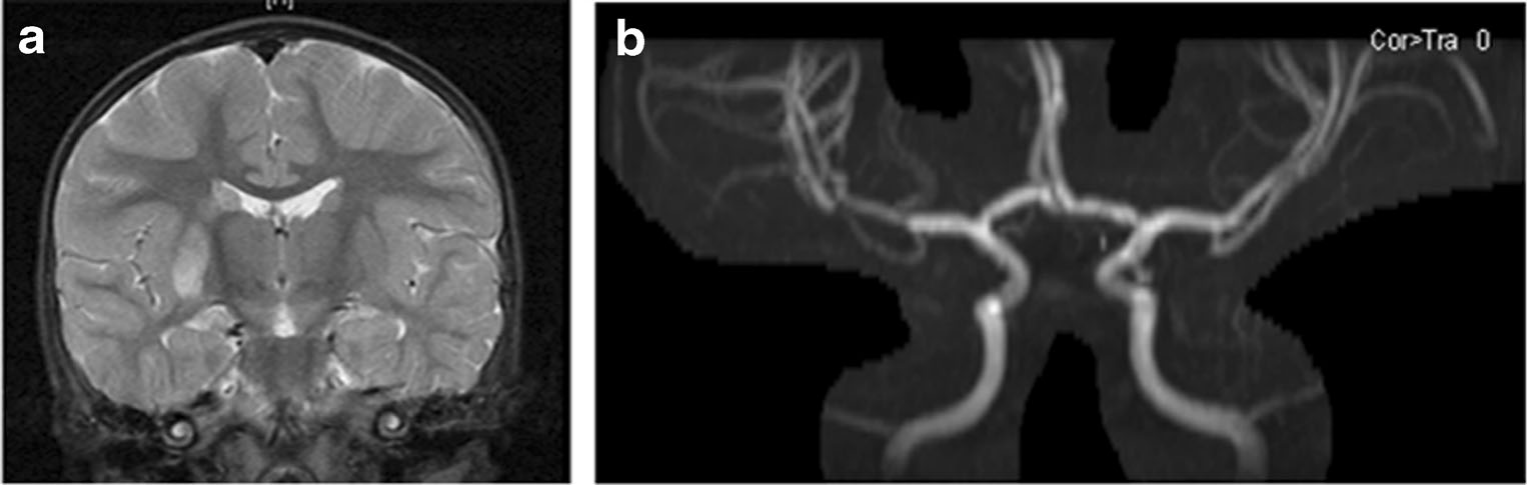
Case 1 illustrates the phenotype of varicella-associated AIS. Brain imaging from a 17 month old boy who presented with an acute L hemiparesis, having had chickenpox 8 months earlier. (**a**) coronal FLAIR images showing high signal in the basal ganglia (caudate and lentiform nuclei) on the right. (**b**) 2D time of flight magnetic resonance imaging showing reduced flow in the distal left M1 segment of the middle cerebral artery, extending distally. No other AIS risk factors were identified despite extensive investigation, including echocardiography. A lumbar puncture was not done and the final diagnosis was post-varicella cerebral infarction. He was treated with aspirin and followed-up until the age of 12 years. He did not have any further clinical or radiological events and made an excellent motor recovery with minimal residual L sided motor signs

**Fig. 2:**
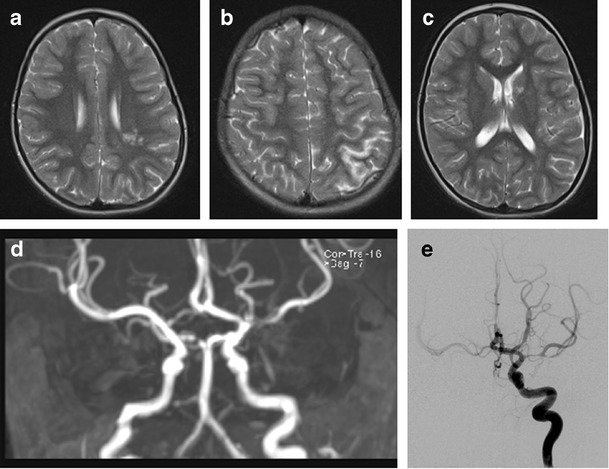
Case 2 illustrates the clinical presentation and imaging findings of varicella vasculitis. Scans from a 7 year old girl who presented with transient weakness of the right arm 3 months after clinical chickenpox. This was her first neurologic presentation. Axial T2 weighted MRI scans (**a**-**c**) show infarcts involving the right periventricular white matter and posterior borderzone region, which had restricted diffusion **(a** and **b**) and an additional lesion with free diffusion in the head of the left caudate (**c**), suggesting an older clinically silent event. 2D time of flight MRA (**d**) showed a focal area of signal drop-out in the M1 segment of the L MCA.CSF was acellular and negative for VZV DNA. A diagnosis of FCA was made and she was treated with aspirin. Three weeks later she presented with a further episode of transient right-sided weakness. Brain imaging did not show any further infarcts but catheter cerebral angiography (**e**) L ICA injection, demonstrated more severe and extensive stenosis of the L MCA. In addition there were bilateral A1 stenoses and unilateral P1 stenosis. CSF examination was repeated; the CSF remained acellular, on this occasion positive for VZV DNA low titer, and VZV IgG was demonstrated to be significantly higher in CSF than in serum. She was treated with a short course of oral steroids and 3 months of acyclovir

In a review of children with VZV associated AIS, common features were that these tended to be younger, otherwise healthy children, with a monophasic clinical course (Case 1, Fig. 1). As previously discussed, they have radiological features of a focal, unilateral, proximal occlusive arteriopathy, indistinguishable from FCA. It has not been routine practice to undertake exhaustive microbiological investigation or lumbar punctures in children with AIS, even with a history of prior VZV. It is well recognized that in many of the latter cases VZV DNA is not detected in the CSF [41]; however, data on VZV antibodies or other infectious biomarkers is scant. This is in contrast to the literature on VZV vasculitis, an entity largely described in adults. These patients commonly have recent herpes zoster and present with more diffuse features such as headache; a progressive or recurrent course is common. Angiographic findings are more variable, with a less consistent pattern of changes, often with multifocal involvement and involvement of multiple sizes of vessel (Case 2, Fig. 2). VZV DNA is found in the CSF in about a third but VZV antibodies in the CSF appear to be a more sensitive diagnostic test (positive in >90 %). Steroids and acyclovir are recommended in the treatment of VZV vasculitis but are not as yet routinely advocated in children with post-varicella cerebral infarction.

VZV associated arteriopathy could be caused by productive virus infection in cerebral arteries [42]. Because the interval between the disease and AIS in children can be as long as 6 or more months, reactivation of latent VZV and subsequent infection of brain arteries, or an ongoing process of persistent silent infection in the brain arteries after the primary infection/viremia and direct invasion of the arterial wall by the virus, could explain the association with of AIS. Asymptomatic VZV reactivation has been demonstrated under stressful conditions, even in immunocompetent individuals [43–46] The observation that in children in the intensive care unit setting VZV DNA was more commonly detected in those who had subclinical primary infection has led to speculation that subclinical chickenpox could be associated with lower levels of viremia and associated cellular immunity, predisposing these children to greater risk of reactivation under stress.

An earlier study in which normal and VZV infected cerebral and temporal arteries were analyzed histologically and histochemically, has shown the presence of VZV primarily in the adventitia early in infection and in the media and intima later [47]. This finding supports the theory that after reactivation from ganglia, VZV spreads transaxonally to the arterial adventitia followed by transmural spread of virus. The authors suggested that stroke in VZV arteriopathy may result from changes in arterial caliber and contractility produced in part by abnormal accumulation of smooth muscle cells and myofibroblasts in thickened neointima and disruption of the media. Animal studies have identified afferent fibers from trigeminal and dorsal root ganglia to both intracranial and extracranial blood vessels [48], providing an anatomic pathway for possible transaxonal spread of virus from the ganglia following reactivation. In addition, pathologic and virological analyses of cerebral arteries from adult patients who died from VZV vasculopathy have revealed herpes virions, VZV antigen, and VZV DNA in the walls of cerebral arteries [49–51].

Much remains unknown about the role of VZV in childhood AIS and it would appear from the adult VZV vasculitis literature that the current approach to investigation of even children with a clear history of VZV is insufficiently detailed. Our current recommendation would be to undertake comprehensive microbiological investigation (including CSF microscopy, PCR for VZV DNA, oligoclonal bands, and VZV antibodies in blood and CSF) in all children with AIS and a history of VZV. Our current practice is to treat patients with VZV DNA or increased CSF antibody titres with acyclovir and steroids. It is well recognized that VZV infection can be subclinical and it is possible that it has a role in childhood AIS where there is no antecedent history of VZV, but this remains a matter of speculation.

## Other Infections Associated with Childhood AIS

### Viral Infections

AIS is a well-recognized complication of Human Immunodeficiency Virus (HIV); potential mechanisms of infarction are primary arteriopathy caused by direct HIV-1 infection of the arterial wall, or secondary because of opportunistic infections, meningitis, encephalitis, vasculitis, coagulopathy, or cardioembolic events [52–54]. The former issue is likely to gain increasing importance with improvements in the long-term survival of children with vertically transmitted HIV.

Other viruses have been linked to cerebrovascular disease and AIS in the published literature but only in case reports or small case series. Apart from VZV, other herpes viruses have been associated with childhood cerebral arteriopathies, including Epstein Barr virus and Cytomegalovirus (CMV) [54–58]. It has been suggested that CMV plays a role in the pathogenesis of arteriosclerotic plaques in cerebral arteries [57]. EBV has been described as a stimulus for AIS during primary EBV infection in children in case reports, but EBV DNA appears to be infrequently detected in CSF in patients with primary EBV infection, viremia, and neurologic manifestations [55, 58]. Parvovirus B19 has been implicated in childhood AIS as a co-factor in patients with underlying sickle cell disease [59]; in a literature review of 81 adult and pediatric cases of neurologic disease associated with Parvovirus B19 and confirmed by detection of viral DNA or specific antibodies in CSF, AIS accounted for approximately 13 % of all central nervous system (CNS) manifestations and was described, as previously mentioned, more commonly in patients with altered immunity or sickle cell disease with aplastic crisis [60]. Enterovirus has been associated with focal stenosis of the proximal middle cerebral artery and presence of enteroviral RNA in cerebrospinal fluid in a case report [61]. Influenza A virus has also been reported in the context of ischemic stroke in children [62, 63].

### Bacterial Infections

Severe bacterial infections as septicemia or meningitis can lead to cerebral infarction by several mechanisms including activation of the coagulation cascade, septic emboli, vascular tissue injury, and inflammation. In bacterial meningitis, the basal cerebral vessel may be bathed in the purulent exudate, thus, there is direct spread of inflammation. A prospective study of 166 children with perinatal and childhood meningitis identified 14 patients with a concomitant stroke; Salmonella species and Streptococcus pneumoniae were the most common causative agents [64].

Mycoplasma pneumoniae has also been reported in relation to childhood stroke in a number of case reports, in a few cases confirmed by intrathecal production of antibodies to mycoplasma pneumoniae or polymerase chain reaction (PCR) in the CSF [65–69]. Other bacterial infectious agents described in the context of pediatric stroke are Borrelia burgdorferi [70–72] and Hemophilus influenza in the prevaccination era [73, 74], and Chlamydia pneumoniae. Bandaru et al reported on a case-control study at single center examining the role of Chlamydia Pneumoniae infection as a risk factor for AIS in the young, and showed Chlamydia pneumoniae seropositivity (IgG) in 27.5 % of the 120 AIS patients vs 12.5 % of the 120 controls [75]. AIS commonly complicates tuberculosis; in a recent 20-year review of central nervous system manifestations of tuberculosis, one-third of the children studied had evidence of a stroke on neuroimaging [76].

### Other Infections

AIS associated with fungal infections (eg, Aspergillus, Candida albicans, Coccidioides immitis, and Cryptococcus neoformans) are rarely encountered in the immunocompetent population [77], but may be observed in the immunocompromised. Such children eg, with malignancy, after bone marrow transplant, or with HIV are encountered not infrequently in clinical practice in large pediatric units.

In summary, although broad categories of infections have been shown to be associated with increased stroke risk, fewer studies have investigated the role of specific pathogens; apart from the case of VZV, the body of evidence for other infectious pathogens (Table 1) comes mainly from case reports with very few exceptions. Recognizing the significance of infections in the development of cerebral arteriopathies and AIS, an international collaboration has led to a prospective study investigating the vascular effects of infection in pediatric stroke (VIPS). The VIPS study, which has recently finished recruiting, will measure association between markers of infection and cerebral arteriopathy and will assess whether cerebral arteriopathy and inflammatory markers predict recurrent stroke [78].

**Table 1:** Infectious agents in childhood stroke and diagnosis

Infectious agent	Specific CSF investigations
Varicella zoster virus	VZV DNA, intrathecal antibodies
Herpes simplex virus type-1	HSV-1 DNA, intrathecal antibodies
Epstein Barr virus	EBV DNA, intrathecal antibodies
Cytomegalovirus	CMV DNA, intrathecal antibodies
Enterovirus	Enteroviral RNA
Mycoplasma pneumoniae	Intrathecal antibodies, polymerase chain reaction
Parvovirus B19	Intrathecal antibodies, polymerase chain reaction
Borrelia burgdorferi	Intrathecal antibodies
Influenza virus	Polymerase chain reaction
Mycobacterium tuberculosis	Culture, immunoblotting, polymerase chain reaction
Hemophilus influenzae	Polymerase chain reaction, intrathecal antibodies
Chlamydia pneumoniae	Intrathecal antibodies, polymerase chain reaction
Salmonella, Streptococcus pneumoniae - meningitis	Culture, polymerase chain reaction (16 s PCR)

## The Role of Trauma in Childhood Arterial Ischemic Stroke

As discussed in the introduction, several single center studies identified prior head and neck trauma in association with childhood AIS. Initial observations were met with a degree of skepticism about whether trauma was the precipitant of AIS or reflected its motor sequelae. Compelling recent, population based, data is from the previously mentioned Northern Californian study, which showed that head/neck trauma in the previous 12 weeks was prevalent and strongly associated with AIS [79]. Multiple potential mechanisms are implicated. Picking up on the theme between genetic predisposition and environmental precipitant, experimental models of some monogenic diseases associated with childhood stroke, specifically mutations in *COL4A1* and neurofibromatosis type 1 suggest that trauma is a necessary cofactor to the genetic predisposition in order to produce stroke. The disease pathways implicated relate to abnormal vessel wall integrity, smooth muscle proliferation or response to injury [3]. As previously discussed trauma is also a potent instigator of an inflammatory response.

Several specific clinical phenotypes link trauma and childhood AIS. One is that of basal ganglia infarction in young children after minor head injury. This is a distinctive phenotype in clinical practice, with a monophasic course and largely favorable outcomes [80–82]. It has been postulated that the underlying mechanism is that of vasospasm of the lenticulostriate perforators for, as yet, unidentified reasons. A recent series has described a group of children with minor head trauma, basal ganglia infarction and evidence of a mineralizing angiopathy of the lenticulostriate perforators apparent on CT [80, 81]. It is intriguing to speculate why such cases have not been widely reported before; one possible explanation is that most children with AIS are now generally investigated with magnetic resonance imaging rather than CT and of childhood AIS cases overall, this group represents a small minority. Several mechanisms have been postulated, for example mechanical stretching of the mineralized lenticulostriate arteries during trauma, a pre-existing neonatal lenticulostriate vasculopathy and congenital or very early childhood infections (CMV, EBV, Mycoplasma) as precursors of mineralization, suggesting that trauma may not be a cause, but a trigger for occlusion of predisposed vessels [81, 82].

The relationship between trauma and cervical arterial dissection is well-recognized [11, 83, 84]. However, in clinical practice, many cases are nontraumatic. A number of patients have underlying inherited connective tissue disorders including Ehlers-Danlos syndrome (type IV), homocystinuria, arterial tortuosity syndrome, and occasionally Marfan syndrome and pseudoxanthoma elasticum [85]. However, a larger group of patients have evidence of more subtle degrees connective tissue abnormality, apparent only on electron microscopy of skin [86, 87]. In addition, infection has been shown to be a risk factor for arterial dissection, another mechanism linking infection and arteriopathy [88].

Intracranial arterial dissection is a more controversial diagnostic entity. At a radiological level, particularly with MRA, published cases are indistinguishable from FCA but in a few such cases there has been histologic evidence of intracranial dissection [89]. Even on catheter angiography pathognomonic features (dissection flap, pseudoaneurysm) may not always be apparent.

## Conclusions

Childhood stroke is an important cause of long-term neurologic morbidity in the young. In contrast to adults, risk factors and disease mechanisms for arterial ischemic stroke are diverse. Current hypotheses about causation suggest interaction between host predisposition (possibly genetically mediated) and secondary insults, often environmental. Of these, infection and trauma are associated, probably by their effect on the cerebral circulation. Current research is focused on examining these interactions in large groups but also on the role of specific pathogens like Varicella Zoster virus. Such work is likely to impact on prevention, treatment and outcome.
